# LncRNA-DUXAP8 Regulation of the Wnt/β-Catenin Signaling Pathway to Inhibit Glycolysis and Induced Apoptosis in Acute Myeloid Leukemia

**DOI:** 10.4274/tjh.galenos.2021.2020.0769

**Published:** 2021-12-07

**Authors:** Hong Zhai, Junting Zhao, Juan Pu, Pan Zhao, Jin Wei

**Affiliations:** 1 The Affiliated Hospital of North Sichuan Medical College, Department of Hematology, Nanchong, China

**Keywords:** Acute myeloid leukemia, LncRNA-DUXAP8, Apoptosis, Glycolysis, Wnt/β-catenin signaling pathway

## Abstract

**Objective:**

Acute myeloid leukemia (AML) is a malignancy of the hematopoietic system, accounting for approximately 70% of acute leukemias. Long noncoding RNA-DUXAP8 (lncRNA-DUXAP8) has been found to be abnormally expressed in a variety of tumors. However, its function and mechanism in AML have not been studied. We investigate the effect of lncRNA-DUXAP8 on AML and its mechanism so as to provide a new theoretical basis for the diagnosis and treatment of AML.

**Materials and Methods:**

The expression of lncRNA-DUXAP8 in AML bone marrow tissues and the THP-1, HL-60, TF-1, AML193, and U937 cell lines was detected by qRT-PCR. It was then altered by transfecting plasmids overexpressing si-DUXAP8 and lncRNA-DUXAP8, respectively. CCK8 and cell colony assay were performed to evaluate the proliferation ability of AML cells. In addition, flow cytometry was used to observe the apoptosis process. Glucose and lactate kits were utilized to detect glucose consumption and lactate levels. Finally, western blotting was performed to detect the expression of proteins related to the Wnt/β-catenin signaling pathway in cells.

**Results:**

LncRNA-DUXAP8 was downregulated in both AML bone marrow tissues and cell lines. Upon interfering with lncRNA-DUXAP8 in AML cell line THP-1, AML cell proliferation and glycolysis were promoted while cell apoptosis was inhibited. The opposite results were obtained after overexpressing lncRNA-DUXAP8. Meanwhile, western blotting confirmed that interference with lncRNA-DUXAP8 stimulated the expression of proteins Wnt5a, β-catenin, c-Myc, and cyclin-D1 in the Wnt/β-catenin pathway. Moreover, overexpression of lncRNA-DUXAP8 inhibited the expression of Wnt/β-catenin pathway proteins. Finally, LiCl, an activator of the Wnt/β-catenin pathway, reversed the regulation of AML cells by lncRNA-DUXAP8 upregulation compared with the DUXAP group.

**Conclusion:**

This study showed that lncRNA-DUXAP8 regulated the Wnt/β-catenin signaling pathway to inhibit glycolysis and induce apoptosis in AML. This experiment has provided new angles and an experimental basis for treating patients with AML.

## Abstract (tr)

**Amaç:**

Akut miyeloid lösemi (AML), hematopoietik sistemin bir malinitesidir ve akut lösemilerin yaklaşık %70’ini oluşturur. Uzun kodlamayan RNA-DUXAP8’in (lncRNA-DUXAP8) çeşitli tümörlerde anormal şekilde ifade edildiği bulunmuştur. Ancak, AML’deki işlevi ve mekanizması çalışılmamıştır. AML’nin tanı ve tedavisi için yeni bir teorik temel sağlamak amacıyla lncRNA-DUXAP8’in AML ve mekanizması üzerindeki etkisini araştırdık.

**Gereç ve Yöntemler:**

AML kemik iliği dokularında ve THP-1, HL-60, TF-1, AML193 ve U937 hücre dizilerinde lncRNA-DUXAP8 ifadesi qRT-PCR ile tespit edildi. Daha sonra sırasıyla si-DUXAP8 ve lncRNA-DUXAP8’i aşırı ifade eden plazmitlerin transfekte edilmesiyle değiştirildi. AML hücrelerinin çoğalma yeteneğini değerlendirmek için CCK8 ve hücre kolonisi testi yapıldı. İlaveten, apoptoz sürecini gözlemlemek için akım sitometri kullanıldı. Glukoz tüketimini ve laktat düzeylerini saptamak için glukoz ve laktat kitleri kullanıldı. Son olarak, hücrelerde Wnt/β-katenin sinyal yolu ile ilgili proteinlerin ifadesini saptamak için western blot testi yapıldı.

**Bulgular:**

LncRNA-DUXAP8, hem AML kemik iliği dokularında hem de hücre dizilerinde baskılanmıştı. AML hücre dizisi THP-1’de lncRNA-DUXAP8’e müdahale edilmesi üzerine, hücre apoptozu inhibe edilirken AML hücre proliferasyonu ve glikoliz kolaylaştırıldı. lncRNA-DUXAP8’ in aşırı ifadesinden sonra tersi sonuçlar elde edildi. Bu arada, western blot yöntemi, lncRNA-DUXAP8 ile etkileşimin Wnt/β-katenin yolağında Wnt5a, β-katenin, c-Myc ve siklin-D1 proteinlerinin ifadesini stimüle ettiğini doğruladı. Ayrıca, lncRNA-DUXAP8’in aşırı ifadesi, Wnt/β-katenin yolu proteinlerinin ifadesini inhibe etti. Son olarak, Wnt/β-katenin yolunun bir aktivatörü olan LiCl, DUXAP grubuyla karşılaştırıldığında lncRNA-DUXAP8 upregülasyonu ile AML hücrelerinin regülasyonunu tersine çevirdi.

**Sonuç:**

Bu çalışma, lncRNA-DUXAP8’in, AML’de glikolizi inhibe etmek ve apoptozu indüklemek için Wnt/β-katenin sinyal yolunu düzenlediğini gösterdi. Bu deney, AML’li hastaları tedavi etmek için yeni açılar ve deneysel bir temel sağlamıştır.

## Introduction

Acute myeloid leukemia (AML) is an aggressive malignancy of the hematopoietic system caused by the abnormal proliferation of myeloid cells in the bone marrow and other hematopoietic tissues [1,2]. AML might occur at any age and accounts for 15%-20% of pediatric leukemias and 80% of adult leukemias [3]. In recent years, breakthroughs have been made in treatment strategies such as intensive chemotherapy and even hematopoietic stem cell transplantation. However, the overall clinical prognosis of AML, especially in elderly patients, generally remains disappointing [4,5]. Poor clinical prognosis of AML is closely related to drug resistance, recurrence, comorbidities, and/or treatment-related mortality in the early stage of chemotherapy [6]. Based on the highly heterogeneous characteristics of AML clones and subclones, a large number of clinical and experimental studies have identified a wide range of AML molecular biological expression profiles, which in turn may facilitate prediction and improvement of the prognosis of patients [7]. Results suggest that analyzing AML molecular heterogeneity and screening corresponding specific molecular targets are significant steps in evaluating the clinical prognosis of AML and developing therapeutic strategies [8].

Long noncoding RNAs (lncRNAs) are a class of endogenous RNAs more than 200 nucleotides in length that are completely lacking or have extremely weak polypeptide coding capacity [9]. Numerous studies have confirmed that lncRNAs located in the nucleus are mainly involved in biomacromolecule-chromatin interactions and transcriptional regulation, as well as RNA processing. Furthermore, lncRNAs located in the cytoplasm can affect not only mRNA stability and translation but also numerous cell signaling pathways [10]. Recent reports have found that lncRNA-HOTAIRMI, which is transcribed from the human HOXA gene cluster, is able to control myeloid cell development by regulating retinoic acid-induced expression of the HOXA1 and HOXA4 genes during bone marrow formation [11]. Some lncRNAs play a key role not only during normal hematopoiesis but also during leukemogenesis [12,13]. One known as lncRNA-HOTAIR exerts a carcinogenic effect by mediating cell proliferation or apoptosis via regulation of c-Kit expression, and it may serve as a biological marker of AML prognosis [14]. Studies have shown that in elderly AML patients with normal cytogenetics, the abnormal expression profile of lncRNAs is closely related to clinical characteristics and the abnormal expression of some lncRNAs is closely related to the curative effect and survival time [15].

Recent studies have reported that lncRNA-DUXAP8 (double homeobox A pseudogene 8) with a length of 2107 bp is located on chromosome 22q11. It is abnormally highly expressed in a variety of tumor tissues and can promote the proliferation of hepatocellular carcinoma [16], bladder cancer [17], pancreatic cancer [18], renal cell carcinoma [19], neuroglioma cells [20], and non-small-cell lung cancer [20]. LncRNA-DUXAP8 is also closely related to the poor prognosis of these tumors. When analyzing pathological data in the clinic, it was found that lncRNA-DUXAP8 was significantly highly expressed in gastric cancer samples with high TNM grades and lymph node metastasis. It was shown that lncRNA-DUXAP8 was closely related to the prognosis of patients, as well, confirming that lncRNA-DUXAP8 may play an important role in the development of gastric cancer as a molecular target for diagnosis and prognosis [21]. The above results suggest that lncRNA-DUXAP8, as an oncogene, can promote the proliferation and invasion of malignant tumor cells and inhibit apoptosis. Therefore, we hypothesize that lncRNA-DUXAP8 is also able to regulate the development of AML, although its specific function and mechanism of action need to be further explored. In this study, we investigate the effect of lncRNA-DUXAP8 (henceforth DUXAP8) on AML and its mechanism so as to provide a new theoretical basis for the diagnosis and treatment of AML.

## Materials and Methods

### Specimen Collection and Disposal

Bone marrow tissues from patients diagnosed with AML and normal bone marrow tissues from healthy donors in our hospital from January 2016 to June 2018 were collected. The clinicopathological characteristics and laboratory features (age, gender, karyotype, etc.) were recorded. The diagnostic and staging criteria used for all patients with first diagnosed leukemia were the 2016 World Health Organization staging criteria [22]. In addition, all patients had not been treated with chemotherapy or radiotherapy. All subjects had no history of major systemic disease and they voluntarily signed informed consents. The study was approved by the Ethics Committee of the Affiliated Hospital of North Sichuan Medical College.

### Cell Culture

Human normal bone marrow cells (HS-5) and five AML cell lines (THP-1, HL-60, TF-1, AML193, and U937) were purchased from the American Type Culture Collection (ATCC, Manassas, VA, USA). All cells were cultured in DMEM medium (Gibco, Waltham, MA, USA) with 10% fetal bovine serum (FBS; Gibco) and 1% penicillin/streptomycin and placed in an incubator with 5% CO_2_ at 37 °C.

### Cell Transfection

Overexpression vectors carrying DUXAP8 interference or full-length sequences as well as the corresponding unloaded si-NC, si-DUXAP8, vector, and DUXAP were synthesized and cloned by Sangon Biotech Co., Ltd. (Shanghai, China) and were then identified by enzyme digestion and sequencing. THP-1 cells in the logarithmic growth phase were seeded in 12-well plates. When the cell confluence reached 50%-60%, si-NC, si-DUXAP8, vector, and DUXAP were transfected into THP-1 cells according to conventional operational steps using Lipofectamine 2000. The cells were divided into five groups as follows: (1) sham: THP-1 cells without transfection; (2) si-NC: THP-1 cells transfected with negative siRNA; (3) si-DUXAP8: THP-1 cells transfected with DUXAP8 siRNA; (4) vector: THP-1 cells transfected with a negative vector; (5) DUXAP8: THP-1 cells transfected with pcDNA-DUXAP8. After 6 h of transfection, fresh media were replaced to culture cells for subsequent experiments.

### CCK8 Assay

The CCK8 assay was used to detect the level of cell proliferation. After the transfected cells had been cultured for 12 h, they were plated in 96-well plates at a concentration of 1x10^4^ cells/well. After that, 10 µL of CCK8 solution (10% concentration) was respectively added at 24, 48, and 72 h. The optical density at 450 nm was measured with an enzyme-labeling instrument. Six duplicated wells were set at each time point in each group. The experiment was repeated three times.

### Colony Formation Assay

Cells from different groups were respectively inoculated in 6-well plates with 300 cells/well. Afterwards, 2 mL of cultivation liquid was added to each well. Cells were cultured in incubators for 10 days until cell clones were visible and then fixed in 4% paraformaldehyde for 15 min and stained with 1% crystal violet for 4 h. After washing and drying, they were observed and photographed. The experiment was repeated three times.

### Flow Cytometry

The Annexin V-PE/7-AAD double-staining method was utilized to detect cell apoptosis. After the transfected cells had been cultured for another 48 h, they were digested with trypsin and washed with PBS two or three times. Then 500 µL of binding buffer and 5 µL of Annexin V-PE were added to about 5x10^5^ cells. After thorough mixing, 5 µL of 7-AAD staining solution was added and the solution was mixed again. After a 10-min reaction at room temperature in the dark, quantitative detection was performed immediately with a FACScan flow cytometer. The experiment was repeated three times.

### Detection of Glucose Consumption and Lactate Production

Cells in the logarithmic growth phase were seeded in 6-well plates with 1.0x10^5^ cells/well. After the transfected cells were cultured for 24 h, 100 µL of supernatant from each group was collected. Supernatants and standards from both the control group and the experimental group were placed on ice. Afterwards, the cells were digested with trypsin and counted for calibration. For determining the glucose content, glucose standards with 0, 1, 2, 3, 4, and 5 µL were first added, followed by double-distilled water to increase the volume of solution in each EP centrifuge tube to 10 µL. After that, the standard curve was obtained, and then 10 µL of supernatant was taken from the experimental group and the control group. To each EP centrifuge tube was added 1 mL of glucose detection reagent with thorough mixing. Tubes were then placed in a water bath at 37 °C for 10 min and 200 µL of mixed solution was removed and added to an ELISA plate. The glucose concentration in each well was detected at the wavelength of 505 nm with an ELISA analyzer.

The method for determination of lactic acid content was similar to the glucose detection method. First, 0, 1, 2, 3, and 4 µL of lactic acid standard was respectively added to the ELISA plates. Afterwards, the volume of each well was made up to 4 µL with double-distilled water to prepare the standard curve and then 4 µL of supernatant from the experimental group and the control group was respectively mixed with 200 µL of lactic acid detection reagent and added into each EP centrifuge tube. The lactic acid concentration of each well was detected at the wavelength of 530 nm with the ELISA analyzer. The experiment was repeated three times.

### qRT-PCR Assay

Based on published references, primer sequences of GAPDH were designed. Total RNA from tissues and cells was respectively extracted using an RNA extraction kit in order to determine its purity and concentration. RNA was reverse-transcribed into cDNA with the TaKaRa reaction system (Shiga, Japan). Subsequently, cDNA was used as a template for the quantitative real-time polymerase chain reaction (qRT-PCR). The reaction system was programmed as follows: pre-denaturation at 95 °C for 5 min, followed by 40 cycles of 95 °C for 10 s, 60 °C for 40 s, and 74 °C for 30 s. Measurements were performed on an ABI 7500 qRT-PCR instrument (Applied Biosystems, Bedford, MA, USA). The primer sequences used in this study were as follows: DUXAP8, forward: 5’-AGGATGGAGTCTCGCTGTATTGC-3’ and reverse: 5’-GGAGGTTTGTTTTCTTCTTTT-3’. GAPDH, forward: 5’-AGGAAGAGCACAAGGAAGGCA-3’ and reverse: 5’-GGTTGCACATAGACGAGGACT-3’. The relative expression of DUXAP8 was calculated using the 2^-^^∆∆^^CT^ method with GAPDH as an internal reference. The experiment was repeated three times.

### Western Blotting

Transfected THP-1 cells were lysed with cell lysate to isolate total intracellular protein. Protein concentration was determined by the BCA quantitative method. First of all, proteins were separated by 12% polyacrylamide-gel electrophoresis (SDS-PAGE). Subsequently, proteins were transferred onto polyvinylidene fluoride membrane and blocked with 5% skimmed milk for 2 h at room temperature. Afterwards, the membranes were incubated with primary antibodies Wnt5a rabbit polyclonal antibody (ab235966, 1:1000), β-catenin rabbit monoclonal antibody (ab32572, 1:1000), c-Myc rabbit monoclonal antibody (CST9402, 1:1000), and cyclin-D1 rabbit monoclonal antibody (ab16663, 1:1000) overnight at 4 °C. The membranes were washed with TBST three times, followed by incubation with the corresponding secondary antibody for 2 h at room temperature. Finally, the membrane was washed three times with TBST for electrochemical luminescence and color development. Relative protein expression was calculated using β-actin as an internal reference. The experiment was repeated three times.

### Statistical Analysis

Statistical analysis was performed using SPSS 20.0 software (IBM Corp., Armonk, NY, USA). Data were presented as mean ± standard deviation. Comparisons among multiple groups were performed using one-way analysis of variance (ANOVA), while comparisons between two groups were performed with Student’s t-test. Kaplan-Meier analyses were used to analyze the impact of DUXAP8 expression on overall survival (OS). Values of p<0.05 were considered statistically significant.

## Results

### Underexpression of DUXAP8 in AML

**Figure 1:**
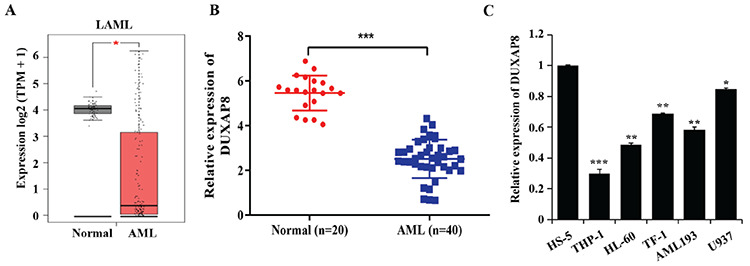
Underexpression of DUXAP8 in acute myeloid leukemia (AML) bone marrow tissues and AML cell lines. (A) The Cancer Genome Atlas database reveals downregulation of DUXAP8 in AML patients; (B) decrease in expression level of DUXAP8 in AML patients’ tissues compared to normal human bone marrow tissues, ***p<0.001: significant difference compared to normal bone marrow; (C) reduction of expression levels of DUXAP8 in AML cell lines THP-1, HL-60, TF-1, AML193, and U937 compared to HS-5 cells, *p<0.05: significant difference compared to HS-5 group, **p<0.01: significant compared to HS-5 group, ***p<0.001: significant compared to HS-5 group.

A retrieval from the Cancer Genome Atlas (TCGA) database revealed that DUXAP8 was downregulated in AML patients (Figure 1A). The results of qRT-PCR confirmed that the expression level of DUXAP8 was significantly reduced in the bone marrow tissues of AML patients compared with normal bone marrow (Figure 1B), which showed that DUXAP8 was involved in the development of AML. To further confirm this, we selected five human AML cell lines, THP-1, HL-60, TF-1, AML193, and U937, as well as normal human bone marrow cell line HS-5 as a control group. qRT-PCR also revealed that the expression of DUXAP8 in AML cell lines THP-1, HL-60, TF-1, AML193, and U937 was obviously decreased compared with HS-5 cells (Figure 1C).

### Low Expression of DUXAP8 Is Associated with Poor Prognosis

**Figure 2:**
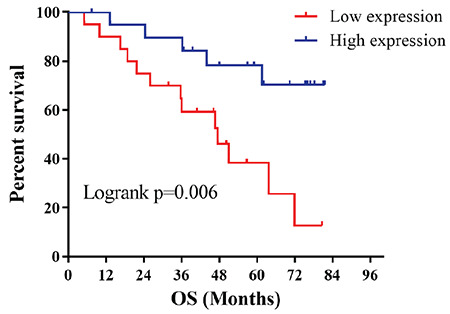
The Kaplan-Meier plots of DUXAP8 expression for overall survival (OS) in acute myeloid leukemia.

The clinical characteristics of DUXAP8-low and DUXAP8-high groups are shown in Table 1. DUXAP8 expression was lower in patients with the AML-M2 subtype (p=0.005) and poor/intermediate risk (p=0.000). Significant differences were also detected for both karyotype and karyotype classification (p=0.000 and p=0.013, respectively). In addition, Kaplan-Meier plots indicated that the OS of the DUXAP8-low group was significantly shorter than that of the DUXAP8-high group (Figure 2).

### DUXAP8 Inhibited Biological Functions in AML Cells

**Figure 3:**
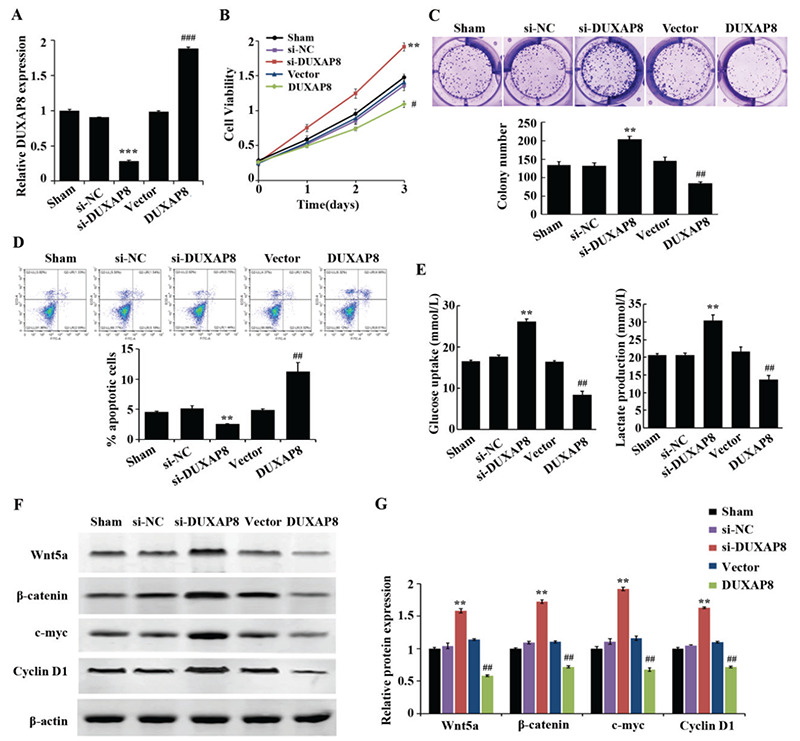
DUXAP8 regulates biological functions of acute myeloid leukemia cells. (A) qRT-PCR was performed to detect DUXAP8 expression level in THP-1 cells after transfection with si-NC, si-DUXAP8, vector, and DUXAP8; (B) CCK8 assay for THP-1 cell proliferation; (C) colony assay for THP-1 cell viability; (D) flow cytometry to evaluate THP-1 apoptosis rate; (E) glucose and lactate kits were utilized to detect glucose consumption and lactate production levels in THP-1 cells, **p<0.01: significant difference compared to si-NC group, ***p<0.001: significant compared to si-NC group, ##p<0.0: significant compared to vector group, ###p<0.001: significant compared to vector group; (F, G) protein expression levels of Wnt5a, β-catenin, c-Myc, and cyclin-D1 were obviously decreased in the cells of the DUXAP8 group compared to the vector group, see previous explanation of statistical significance.

In order to further explore the biological functions exerted by DUXAP8 in AML, DUXAP8 was overexpressed and interfered with. The biological functions of viability, proliferation, and apoptosis of THP-1 cells were then detected using the CCK8 assay, cell colony assay, and flow cytometry, respectively. In addition, glucose consumption and lactate production were detected using a glucose kit and lactate kit. Afterwards, the expression levels of Wnt/β-catenin signaling pathway-related proteins (Wnt5a, β-catenin, c-Myc, cyclin-D1) were verified by western blotting. Since DUXAP8 had the lowest expression in the THP-1 cell line, this cell line was used for subsequent experiments. The results suggested that compared to the si-NC group, the proliferation ability, cell viability, glucose consumption, and lactate production in the si-DUXAP8 group were significantly increased while apoptosis was inhibited. On the contrary, compared with the vector group, the proliferation ability, cell viability, glucose consumption, and lactate production of THP-1 cells were significantly decreased while apoptosis was promoted (Figures 3A-3E). Meanwhile, according to western blotting, the protein expression levels of Wnt5a, β-catenin, c-Myc, and cyclin-D1 were greatly increased in the cells of the si-DUXAP8 group compared to the si-NC group. In contrast, the protein expression levels of Wnt5a, β-catenin, c-Myc, and cyclin-D1 were obviously decreased in the cells of the DUXAP8 group compared to the vector group (Figures 3F and 3G). These experimental results confirmed that overexpression of DUXAP8 inhibited glycolysis and induced apoptosis of AML cells, ultimately inhibiting the activation of the Wnt/β-catenin signaling pathway.

### LiCl as an Activator of the Wnt/β-Catenin Pathway Reversed the Regulation of DUXAP8 in AML Cells

**Figure 4:**
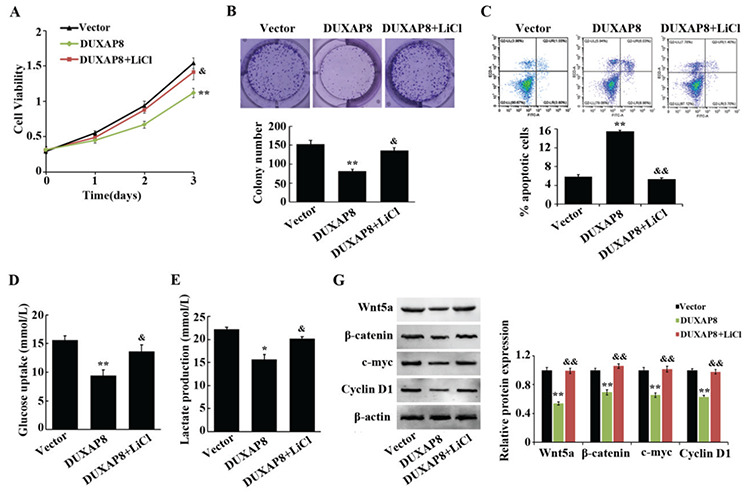
LiCl as an activator of the Wnt/β-catenin pathway reverses the regulation of DUXAP8 on acute myeloid leukemia cells. (A) CCK8 assay to detect the cell proliferation of THP-1 after transfection with vector, DUXAP8, and DUXAP8 + LiCl; (B) cell colony assay to detect the cell viability of THP-1 after transfection with vector, DUXAP8, and DUXAP8 + LiCl; (C) flow cytometry to detect the apoptosis rate of THP-1 after transfection with vector, DUXAP8, and DUXAP8 + LiCl; (D, E) glucose and lactate kits were utilized to detect glucose consumption and lactate production levels of THP-1 after transfection with vector, DUXAP8, and DUXAP8 + LiCl, *p<0.05: significant difference compared to the vector group, **p<0.01: significant compared to the vector group, ^&^p<0.05: significant compared to the DUXAP8 group, ^&&^P<0.01: significant compared to the DUXAP8 group; (F, G) protein expression levels of Wnt5a, β-catenin, c-Myc, and cyclin-D1 were significantly increased in THP-1 cells, see previous explanation of statistical significance.

The above experimental results confirmed that the biological functions exerted by DUXAP8 in AML are directly related to the Wnt/β-catenin pathway. In order to further clarify its molecular mechanism, we not only overexpressed DUXAP8 in THP-1 cells but also added the Wnt/β-catenin pathway activator LiCl. The results showed that compared with the DUXAP8 group, the proliferation level, cell viability, glucose consumption, and lactate production level of cells in the DUXAP8 + LiCl group were greatly increased while apoptosis was effectively inhibited (Figures 4A-4E). Meanwhile, the protein expression levels of Wnt5a, β-catenin, c-Myc, and cyclin-D1 were significantly increased in THP-1 cells (Figures 4F and 4G). These experimental results confirmed that DUXAP8 may inhibit AML cell function by regulating the activation of the Wnt/β-catenin pathway.

## Discussion

Numerous reports have pointed out that lncRNA plays an oncogene role in the development of tumors by regulating cancer cell metastasis and cell growth as well as inhibiting cancer apoptosis, or by interacting with other genes to cause DNA damage [23,24]. Recent studies have found that lncRNA as an antisense gene locus in PU.1 can negatively regulate the expression of hematopoietic transcription factor PU.1 to maintain normal hematopoietic development, thereby inhibiting leukemia [25]. In addition, antisense lncRNA-IRAIN, which is transcribed from the insulin-like growth factor type I receptor serving as the gene locus, plays a negative regulatory role in high-risk AML patients [26]. Previous studies have revealed that DUXAP8 promotes bladder cancer cell proliferation by regulating PTEN [17]. By downregulating DUXAP8 in lung cancer cells, it is not only possible to inhibit the proliferation of pancreatic cancer cells through silencing CDKN1A and KLF2, but also to induce their apoptosis [18]. However, its biological functions and regulatory mechanism in AML are still unclear. In this study, it has been demonstrated by the TCGA database and qRT-PCR assay that DUXAP8 is expressed at lower levels in AML, and AML patients with low DUXAP8 expression showed worse prognosis. On the basis of this result, we then investigated the effects as well as the specific mechanism of DUXAP8 on biological characteristics of AML cells.

Rapid proliferation and metastasis of tumor cells require much energy and, therefore, energy metabolism is of great importance [27,28]. The mainstream view is that tumor cells consume large amounts of glucose to supply tumor cells via glycolysis under oxygen-sufficient conditions, known as the classical Warburg effect [29]. To date, glycolysis is still widely considered as an energy source for tumor cells [30]. In the present study, it was suggested that the glucose consumption and lactate production levels in AML THP-1 cells increased significantly after interference with DUXAP8 expression, while they decreased significantly with overexpression. This result implied that DUXAP8 was able to effectively inhibit glycolysis in AML. It is well known that abnormal proliferation and apoptosis are among the markers of tumor cells. Therefore, the core of antitumor methods is to inhibit the proliferation of tumor cells and promote their apoptosis [31,32]. In subsequent experiments, we applied the CCK8 assay, cell colony assay, and flow cytometry to respectively detect AML cell proliferation, cell viability, and apoptosis. The results showed that cell proliferation and viability were significantly promoted and apoptosis was inhibited in AML cells after interfering with DUXAP8. Opposite results were displayed after overexpressing DUXAP8. Thus, DUXAP8 might inhibit the proliferation of AML cells as well as promoting their apoptosis.

After identifying the effect of DUXAP8 on the biological characteristics of AML, it is necessary to focus on its specific mechanism in subsequent experiments. Various reports have confirmed that many signaling pathways (e.g., the mTOR signaling pathway) play key roles in all kinds of cellular processes, including the regulation of gene expression, cell growth, and proliferation. Studies have found that cell proliferation and metastasis are important factors for cancer development. It should be noted that the Wnt/β-catenin signaling pathway is a crucial signaling pathway for regulation of cell proliferation and metastasis, often abnormally activated in the development of many cancers including prostate cancer, oral squamous cell carcinoma, and cutaneous squamous cell carcinoma [33,34,35]. Additional studies also confirmed that the Wnt/β-catenin signaling pathway is able to accelerate the glycolytic process so as to further provide energy for tumor cells [36,37]. Previous studies have confirmed that DUXAP8 inhibits biological function by inhibiting proliferation and glycolysis while promoting the apoptosis of AML cells. Therefore, this signaling pathway may be a target pathway. Since Wnt5a, β-catenin, c-Myc, and cyclin-D1 are key factors in the Wnt/β-catenin signaling pathway [38], it is essential to increase their expressions for Wnt/β-catenin activation [39]. All in all, activation of this signaling pathway contributes to promoted survival of tumor cells, which leads to unlimited proliferation during the tumor phase.

In this study, western blotting was performed to detect the correlation between DUXAP8 and the Wnt/β-catenin signaling pathway. The results of the western blot assay showed that upon interfering with the expression of DUXAP8, expression levels of Wnt5a, β-catenin, c-Myc, and cyclin-D1 were upregulated; however, after overexpressing DUXAP8, these expression levels were significantly downregulated. In addition, the upregulation of Wnt5a, β-catenin, c-Myc, and cyclin-D1 indicated that the Wnt/β-catenin signaling pathway was inhibited. Therefore, it was speculated that DUXAP8 might exert biological functions in terms of inhibiting glycolysis and inducing apoptosis in AML by inhibiting the activation of the Wnt/β-catenin signaling pathway. Subsequent experiments further validated this speculation. By adding LiCl to THP-1 cells while overexpressing DUXAP8, the biological function of the AML cell lines was detected. These results confirmed that LiCl as an activator of the Wnt/β-catenin pathway reversed the regulation of DUXAP8 in AML cells. These results suggest that DUXAP8 might inhibit glycolysis and induce apoptosis in AML by regulating the Wnt/β-catenin signaling pathway.

## Conclusion

The current study found that DUXAP8 was expressed at lower levels in AML. DUXAP8 was able to inhibit the activation of the Wnt/β-catenin signaling pathway and weaken the proliferation, viability, and glycolytic process of AML cells, as well as exacerbating apoptosis. These results suggest that DUXAP8 plays an important role in AML development and may become a therapeutic target for AML. However, there are some shortcomings of this study; in particular, the functional mechanism of DUXAP8 was not verified in animals. This will need to be further explored in subsequent studies.

## Figures and Tables

**Table 1:**
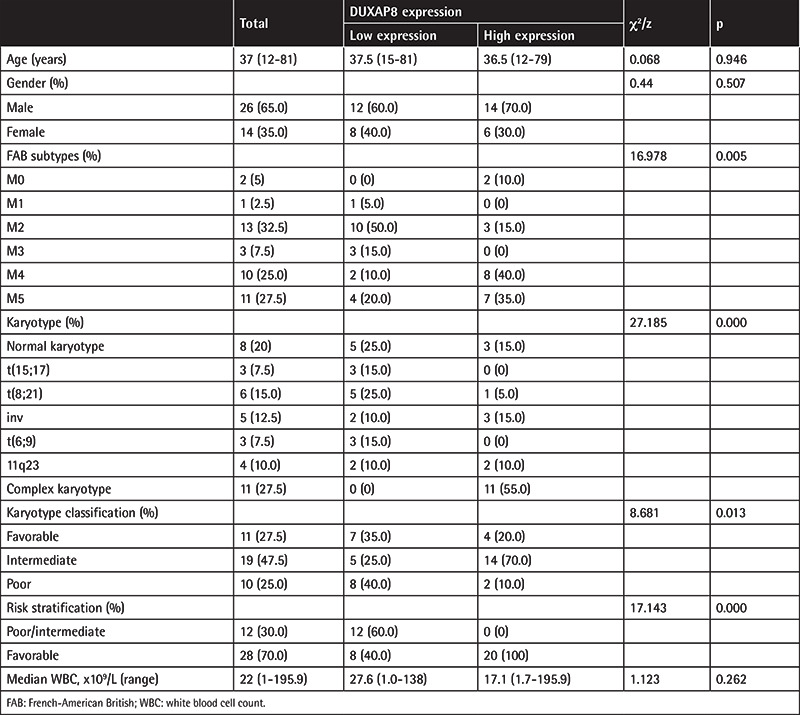
Comparison of clinical and genetic characteristics of patients with AML.
